# Burden trends and disparities of metabolic risk-attributable peripheral arterial disease in China (1990–2035): a comparative analysis with G20 countries

**DOI:** 10.3389/ijph.2026.1610164

**Published:** 2026-09-15

**Authors:** Jie Yuan, Baolei Guo, Fan Li, Qianchuan Yi, Likuan Tu, Bo Tang, Zhirong Li

**Affiliations:** 1 Department of General Surgery, University-Town Hospital of Chongqing Medical University, Chongqing, China; 2 Department of Vascular Surgery, Zhongshan Hospital, Fudan University, Shanghai, China; 3 Institute of Vascular Surgery, Fudan University, Shanghai, China; 4 Department of Surgery and Anesthesiology, University-Town Hospital of Chongqing Medical University, Chongqing, China

**Keywords:** China, G20 countries, GBD 2023, metabolic risks, peripheral arterial disease

## Abstract

**Objective:**

Peripheral arterial disease (PAD) presents a significant clinical and public health challenge. This study aimed to comprehensively analyze and compare the burden trends and disparities of PAD attributable to metabolic risks in China and G20 countries.

**Methods:**

Disability-adjusted life years (DALYs), age-standardized rates, population-attributable fractions, and average annual percentage changes (AAPCs) were used as the burden metrics. Decomposition analysis was conducted to quantify the contributions of population growth, aging, and epidemiological changes. The autoregressive integrated moving average (ARIMA) model was applied to project future burden trends.

**Results:**

In 2023, China and G20 countries had 100 298 and 704 214 DALYs of PAD attributable to metabolic risks, respectively. Compared to 1990, these figures increased by 210.9% and 114.6%, respectively. Population growth and aging were major drivers of the increasing PAD burden. Significant disparities were observed across ages and sexes. Moreover, projections indicate an increasing DALYs in China and G20 countries by 2035.

**Conclusion:**

Our findings reveal a substantial and growing absolute PAD burden attributable to metabolic risks in China and G20 countries, with China exhibiting a distinct epidemiological pattern.

## Introduction

Lower extremity peripheral arterial disease (PAD) is a common cardiovascular disease characterized by atherosclerotic arterial stenosis or occlusion in the lower limbs. It is significantly associated with impaired quality of life, elevated amputation risk, and major adverse cardiovascular events [1–3]. Globally, PAD affects more than 200 million people, with an estimated prevalence of 1.52%. This prevalence increases significantly with age, reaching 20.70% in individuals aged 90–94 years [4, 5]. Driven by population growth and aging, the number of prevalent cases worldwide is projected to increase by 220%, reaching 360 million by 2050 [6]. Notably, substantial evidence highlights a strong association between metabolic risks and PAD [1, 2]. From 2010 to 2023, the metabolic risk-attributable burden showed a significant increasing trend globally, particularly among older adults [7].

As the country with the largest elderly population globally, China is facing further aging challenges with “second baby boomers” (born between 1962 and 1975, the second major post-1949 demographic birth peak, whose members are now entering older adulthood) beginning to retire [8, 9]. Moreover, the rapid dietary transition, lifestyle changes, and accelerated urbanization in China have significantly increased the prevalence of metabolic diseases [10, 11]. The combined effects of population aging and metabolic risks have led to a persistent increase in the absolute burden of PAD in China, which poses a significant challenge to the public health system [12, 13]. The Group of Twenty (G20) represents the largest economy globally and accounts for approximately 85% of the global GDP and almost two-thirds of the world’s population and land area, indicating its significant representativeness and influence [14, 15]. Therefore, conducting a comparative analysis of the disease burden with that of the G20 countries has important implications for China in formulating effective strategies and optimizing healthcare resource allocation.

However, research on the PAD burden attributable to metabolic risks remains limited, particularly concerning China and the G20 countries. Most prior studies were based on the Global Burden of Disease (GBD) 2021, highlighting the need for updated analyses. The recently released GBD 2023 study, which provides comprehensive estimates for 375 diseases and 88 risk factors across 204 countries and territories from 1990 to 2023, enables an assessment of the latest disease burden trends [7]. This study aimed to utilize the latest GBD 2023 data to comprehensively analyze and compare the metabolic risk-attributable PAD burden and its temporal trends in China and G20 countries. Additionally, we quantified the contributions of population growth, aging, and epidemiological changes to changes in the PAD burden and projected future trends by 2035. Our findings are expected to improve the understanding of the epidemiology of the metabolic risk-attributable PAD burden in China, support evidence-based public health strategies, and reduce the growing burden of PAD on China’s public health systems.

## Methods

### Data sources

Since 1990, GBD studies have systematically quantified health and health loss across age, sex, location, and year. In GBD 2023, PAD was defined as an ankle-brachial index (ABI) ≤ 0.90, with corresponding ICD-10 codes (I70.2-I70.8, I73-I73.9) [16]. We retrieved data on the disability-adjusted life years (DALYs) of PAD attributable to all metabolic risks and their corresponding uncertainty intervals (UIs) in China and G20 countries from 1990 to 2023. The G20 countries included China, Canada, Argentina, Türkiye, Australia, Germany, Brazil, France, India, Indonesia, Mexico, Italy, Japan, Republic of Korea, Russian Federation, Saudi Arabia, South Africa, United Kingdom, United States of America, and European Union. All data for this study were obtained from GBD 2023 via the Global Health Data Exchange results tool (https://vizhub.healthdata.org/gbd-results/). The GBD study used anonymized data, and the University of Washington Institutional Review Board granted a waiver of informed consent [17]. This study adheres to the accurate and transparent health estimates reporting (GATHER) guidelines [18].

### Burden metrics

DALYs, age-standardized DALY rates (ASDRs), population-attributable fractions (PAFs), and average annual percentage changes (AAPCs) were used as burden metrics. DALYs are a comprehensive metric for assessing the overall burden of disease and injury. It combines health losses resulting from both fatal and nonfatal outcomes to measure the health status of a population over a specific period, and has been widely adopted by global health institutions, such as the WHO [7]. The age-standardized rate (ASR) represents a weighted average of age-specific rates, eliminating the confounding effect of differences in age distribution between populations. It is calculated via a specific formula based on the GBD standard population structure (Supplementary Material 1.1). The standard population structure used in this study was downloaded directly from the Institute for Health Metrics and Evaluation (IHME) resources, which have been previously validated and widely applied [19–21]. AAPC is a summary measure of the trend over a prespecified fixed interval. It is calculated via a specific formula and remains valid even if the trend changes during this period (Supplementary Material 1.2). If the 95% confidence interval (CI) for AAPC is entirely above zero, it indicates an overall increasing trend; if it is entirely below zero, it indicates an overall declining trend; if the 95% CI contains zero, the trend is considered stable.

### Risk factors

GBD 2023 provides a comprehensive estimate of exposure levels, relative health risks, and attributable burdens for 88 risk factors [7]. In GBD 2023, all risk factors were classified into a risk factor hierarchy with four levels. Metabolic risk factors were classified as level 1 and then disaggregated at level 2 into 6 risk factors, including high fasting plasma glucose, high systolic blood pressure, high body-mass index, kidney dysfunction, high low-density lipoprotein cholesterol, and low bone mineral density (definitions in Supplementary Material 1.3). All analyses were based on the GBD comparative risk assessment framework and employed sophisticated analytical methods, including disease model meta-regression, spatiotemporal Gaussian process regression, and the burden of proof method [7]. These methods generate estimates of the risk factor-attributable burden and clarify the contribution of modifiable risk factors. The population-attributable fraction (PAF) was used to quantify the contribution of metabolic risks, which is the proportional change in health risk that would occur if exposure to a risk factor were reduced to the theoretical minimum risk exposure level. Notably, PAFs for different risk factors are not mutually exclusive, individuals may be exposed to multiple risk factors simultaneously, so the sum of PAFs can exceed 100%. All methods are detailed in the GBD 2023 Collaborator’s report [7].

### Decomposition analysis

Decomposition analysis was conducted to quantify the contributions of population growth, aging, and epidemiological changes to the observed changes in metabolic risk-attributable DALYs. Epidemiological changes reflect the combined effects of all factors other than age structure and population size, including changes in preventive measures, medical technologies, and risk factor exposure. The decomposition results include both the absolute and relative contributions of each factor. The detailed specifications of the decomposition methodology are described in Supplementary Material 1.4 and have been widely established in previous studies [22–24].

### Autoregressive integrated moving average (ARIMA) model

We applied the ARIMA model to project future burden trends by 2035. As a widely applied time series analysis method, this model effectively captures trends and cyclical patterns within time series data and projects future changes based on existing data [25–27]. The ARIMA model combines autoregression (AR), integration (I), and moving average (MA) components. We first applied the differencing method to stabilize the time series data and used the Kwiatkowski-Phillips-Schmidt-Shin (KPSS) test to confirm stationarity. Then, a Q-Q plot was utilized to assess whether the residuals followed a normal distribution. Next, we compared the goodness-of-fit of different models using the Akaike information criterion (AIC) and Bayesian information criterion (BIC), selecting the model with the smallest criterion value. Finally, the Ljung-Box test was used to verify the robustness of the residuals. When the residuals of the ARIMA model exhibit randomness (white noise), the model is considered the best linear predictor for short-term time series forecasting. Separate ARIMA models were independently fitted for males, females, and both-sex aggregated dataset. The both-sex projected DALYs were directly generated from its own ARIMA model, rather than derived by summing sex-specific forecasting outputs. The detailed analysis method is presented in Supplementary Material 1.5.

### Statistical analyses and data visualizations

In this study, we stratified the metabolic risk-attributable PAD burden by age and sex to evaluate disparities. All the statistical analyses and data visualizations were performed via R (version 4.4.2) and JD_GBDR (V2.7.5, Jingding Medical Technology Co., Ltd), and *P* < 0.05 was considered statistically significant.

## Results

### Metabolic risk-attributable PAD burden

In 2023, the total DALYs of PAD were 184 222 (95% UI: 96 253 to 329 430) in China and 1 262 488 (95% UI: 975 578 to 1 692 600) in G20 countries. The attributable DALYs were 124 709 (95% UI: 65 939 to 229 079) and 852 838 (95% UI: 638 987 to 1 193 377), respectively.

Among these, metabolic risk-attributable DALYs were 100 298 (95% UI: 51 116 to 184 384) in China and 704 214 (95% UI: 495 812 to 1 001 461) in G20 countries, accounting for 54.5% and 55.8% of the total DALYs, and 80.4% and 82.6% of the attributable DALYs, respectively (Table 1). From 1990 to 2023, metabolic risk-attributable DALYs increased by 210.9% in China (AAPC: 3.55 [95% CI: 3.46 to 3.65]) and 114.6% in G20 countries (AAPC: 2.41 [95% CI: 2.26 to 2.55]). However, during this period, the ASDR decreased by 11.4% (AAPC: −0.35 [95% CI: −0.46 to −0.25]) in China and 23.8% (AAPC: −0.77 [95% CI: −0.89 to −0.65]) in G20 countries (Table 1).

**TABLE 1:** Number and age-standardized rate (ASR) of Disability-adjusted life years (DALYs) for peripheral arterial disease attributable to risk factors in 1990/2023, and corresponding average annual percentage change (AAPC) from 1990 to 2023. (Chongqing, China. 2026).

Risk factors	Number			ASR per 100000		
	1990 (95% UI)	2023 (95% UI)	1990–2023 AAPC (95%CI)	1990 (95% UI)	2023 (95% UI)	1990–2023 AAPC (95%CI)
China						
Total (all-cause)	70 047 (37 890, 124 576)	184 222 (96 253, 329 430)	3.04 (2.99, 3.10)	10.29 (5.45, 17.70)	8.03 (4.22, 14.34)	−0.72 (−0.78, −0.65)
All risk factors	45 995 (25 058, 83 331)	124 709 (65 939, 229 079)	3.10 (3.02, 3.18)	6.74 (3.69, 12.00)	5.44 (2.90, 9.90)	−0.65 (−0.75, −0.56)
Metabolic risks	32 259 (16 934, 57 802)	100 298 (51 116, 184 384)	3.55 (3.46, 3.65)	4.98 (2.56, 8.91)	4.41 (2.26, 8.09)	−0.35 (−0.46, −0.25)
High fasting plasma glucose	12 447 (6 060, 23 671)	50 442 (25 499, 91 994)	4.39 (4.33, 4.45)	2.03 (0.96, 3.84)	2.23 (1.15, 4.09)	0.33 (0.26, 0.40)
High systolic blood pressure	8 014 (1 446, 19 147)	23 541 (4 906, 51 675)	3.28 (3.22, 3.33)	1.20 (0.21, 2.85)	1.03 (0.21, 2.24)	−0.47 (−0.55, −0.40)
High body mass index	4 096 (929, 11 732)	21 108 (5 029, 57 365)	5.07 (5.01, 5.14)	0.50 (0.11, 1.45)	0.89 (0.21, 2.41)	1.78 (1.70, 1.86)
Kidney dysfunction	18 299 (7 822, 36 535)	52 010 (21 419, 110 349)	3.15 (2.96, 3.33)	2.84 (1.22, 5.43)	2.29 (0.94, 4.80)	−0.63 (−0.83, −0.44)
G20						
Total (all-cause)	656 701 (532 186, 846 082)	1 262 488 (975 578, 1 692 600)	1.93 (1.84, 2.03)	27.53 (22.41, 35.25)	19.47 (15.06, 26.00)	−1.01 (−1.14, −0.88)
All risk factors	439 545 (339 585, 581 610)	852 838 (638 987, 1 193 377)	2.10 (1.94, 2.25)	18.33 (14.13, 24.33)	13.16 (9.89, 18.32)	−0.95 (−1.08, −0.82)
Metabolic risks	328 155 (228 813, 450 441)	704 214 (495 812, 1 001 461)	2.41 (2.26, 2.55)	14.28 (10.04, 19.54)	10.88 (7.70, 15.42)	−0.77 (−0.89, −0.65)
High fasting plasma glucose	126 217 (81 968, 192 993)	341 763 (239 198, 507 668)	3.13 (2.99, 3.27)	5.77 (3.78, 8.60)	5.29 (3.72, 7.85)	−0.25 (−0.37, −0.13)
High systolic blood pressure	87 585 (17 068, 160 012)	157 617 (31 453, 289 358)	1.78 (1.63, 1.94)	3.68 (0.72, 6.74)	2.43 (0.48, 4.47)	−1.22 (−1.37, −1.07)
High body mass index	97 815 (25 842, 221 635)	255 017 (69 488, 537 032)	2.90 (2.77, 3.03)	3.97 (1.06, 9.01)	3.91 (1.07, 8.21)	−0.10 (−0.22, 0.03)
Kidney dysfunction	190 045 (106 492, 292 352)	392 842 (223 296, 624 890)	2.23 (2.10, 2.36)	8.31 (4.69, 12.69)	6.08 (3.46, 9.67)	−0.91 (−1.03, −0.79)

Notably, metabolic risk-attributable DALYs showed an increasing trend across all G20 member countries from 1990 to 2023, with China ranking eighth regarding the growth magnitude. In terms of ASDR, 12 out of 20 G20 members presented declining trends over this period, and China ranked ninth regarding the decline magnitude (Supplementary Table 1).

### Metabolic risk-attributable PAD burden by year and sex

Notable sex disparities in the metabolic risk-attributable PAD burden were observed (Supplementary Table 2; Figure 1). In China, DALYs increased consistently from 1990 to 2023 for both sexes, whereas the ASDR showed a slight fluctuating decline, with consistently higher DALYs and ASDRs in females than in males. During this period, DALYs for both sexes in G20 countries also increased, but the ASDR decline was more pronounced. Although females consistently had higher DALYs than males, their ASDRs were significantly lower than those of males.

**FIGURE 1:**
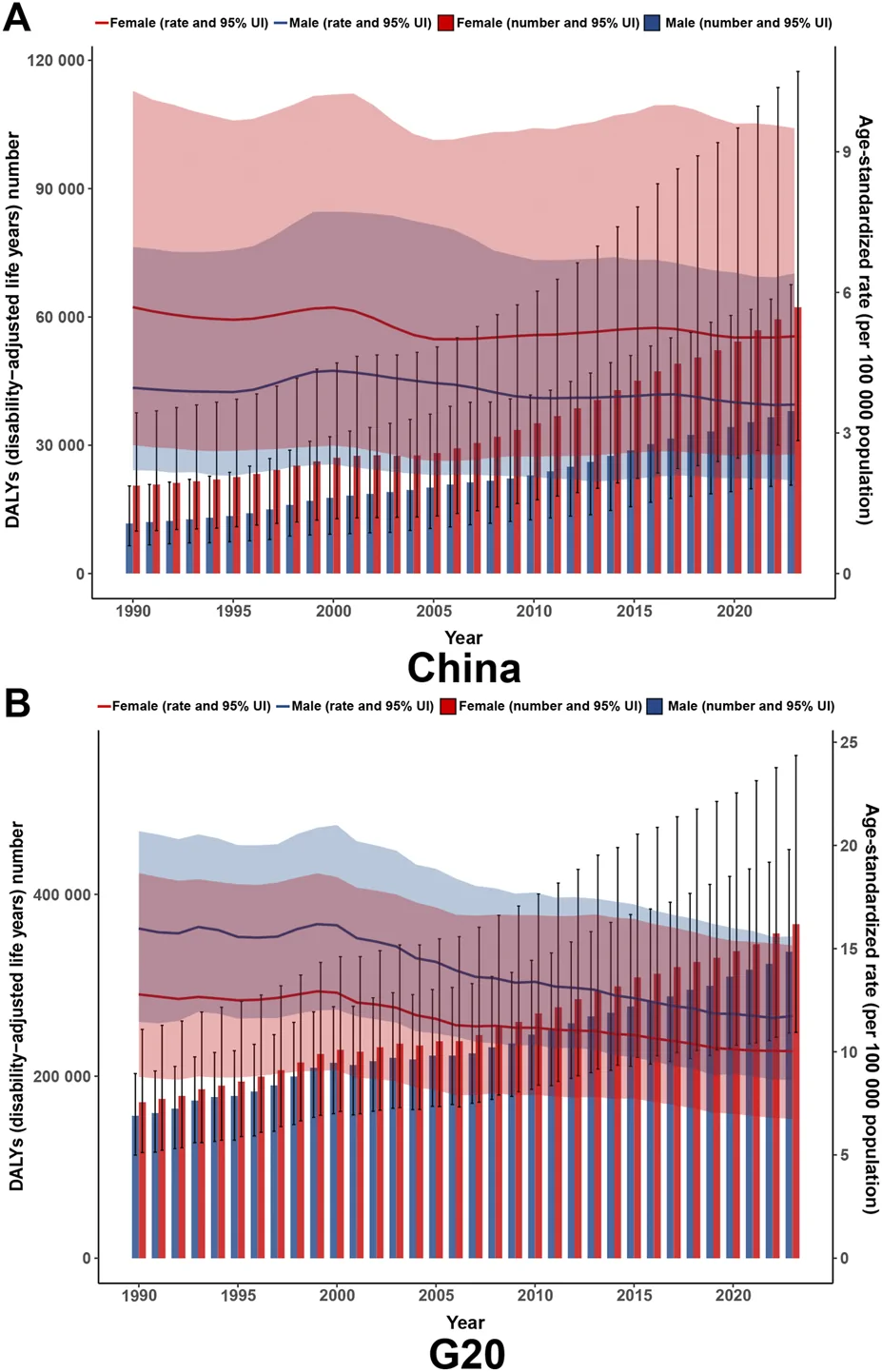
Number and age-standardized rate (ASR) of disability-adjusted life years (DALYs) for peripheral arterial disease attributable to metabolic risks in China **(A)** and G20 countries **(B)** by year and sex. (Chongqing, China. 2026).

### Metabolic risk-attributable PAD burden by age and sex

The metabolic risk-attributable PAD burden exhibited significant age disparities (Supplementary Table 3; Figure 2). In China, DALYs peaked in the 70–74 years group and then declined for both sexes in 2023, whereas the ASDR generally continued to increase with advancing age. Females had significantly higher DALYs and ASDRs in the age groups above 55 years. In G20 countries, DALYs peaked in the 70–74 years group for males and the 75–79 years group for females during this period. Males had higher DALYs and ASDRs in younger age groups, but were surpassed by females above the 75 years group for DALYs and the 85 years group for ASDRs.

**FIGURE 2:**
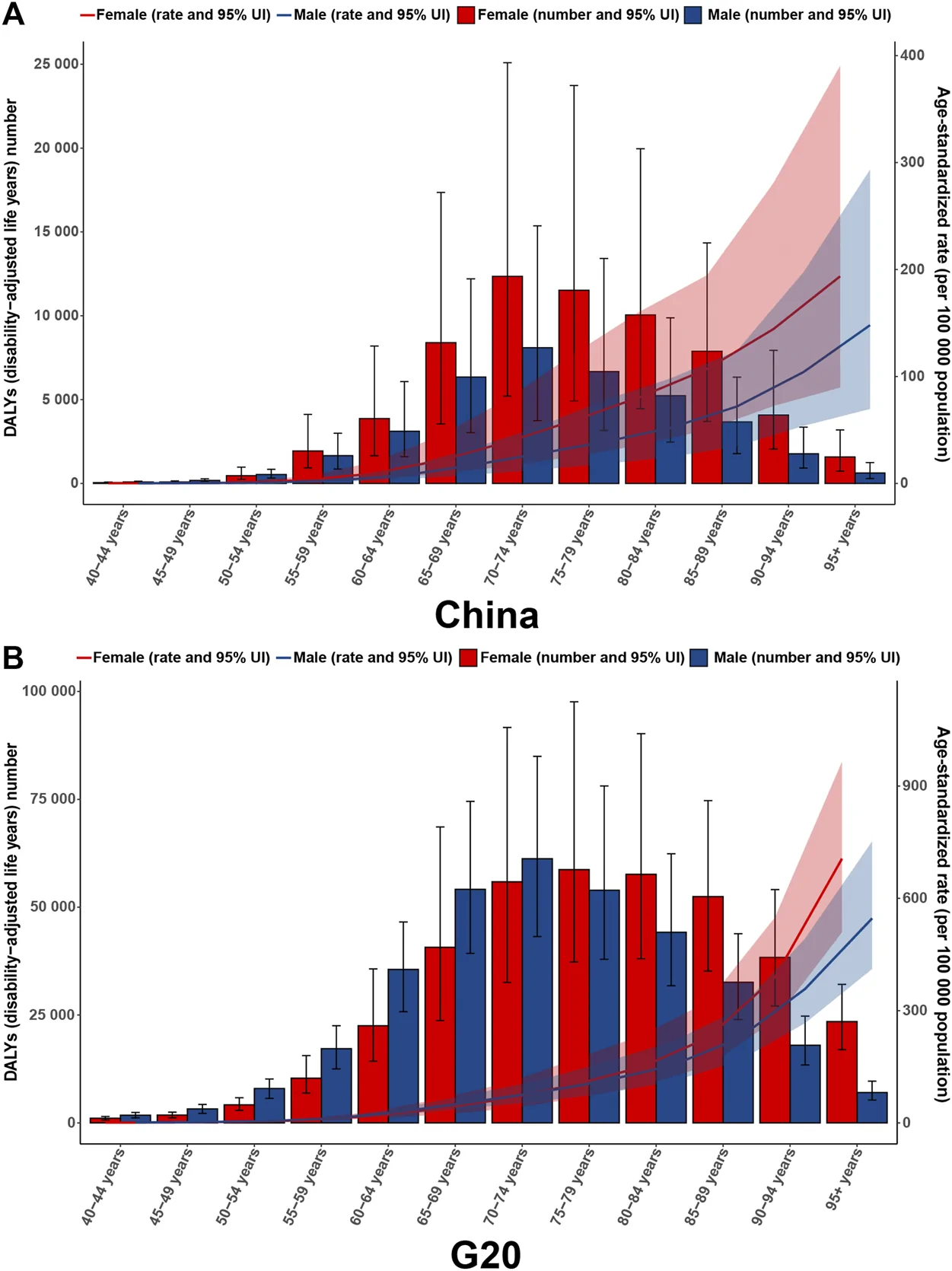
Number and age-standardized rate (ASR) of disability-adjusted life years (DALYs) for peripheral arterial disease attributable to metabolic risks in China **(A)** and G20 countries **(B)** by age group and sex in 2023. (Chongqing, China. 2026).

### Specific metabolic risk factor-attributable PAD burden

High fasting plasma glucose, high systolic blood pressure, high body-mass index, and kidney dysfunction were identified as specific metabolic risk factors for PAD burden in GBD 2023. Kidney dysfunction was the leading metabolic risk factor, accounting for 28.3% of the population-attributable burden in China and 31.1% in G20 countries, followed by high fasting plasma glucose (27.4% in China and 27.1% in G20 countries). High body-mass index (11.5% in China and 20.3% in G20 countries) and high systolic blood pressure (12.9% in China and 12.5% in G20 countries) had relatively lower impacts (Table 1).

From 1990 to 2023, the PAFs for kidney dysfunction and high systolic blood pressure remained relatively stable in both China and the G20 countries. However, the PAFs for high fasting plasma glucose and high body-mass index exhibited a marked increase in both China and the G20 countries, with China experiencing a greater increase than the G20 countries (Figure 3). Similar trends were observed for both sexes (Supplementary Figures 1, 2).

**FIGURE 3:**
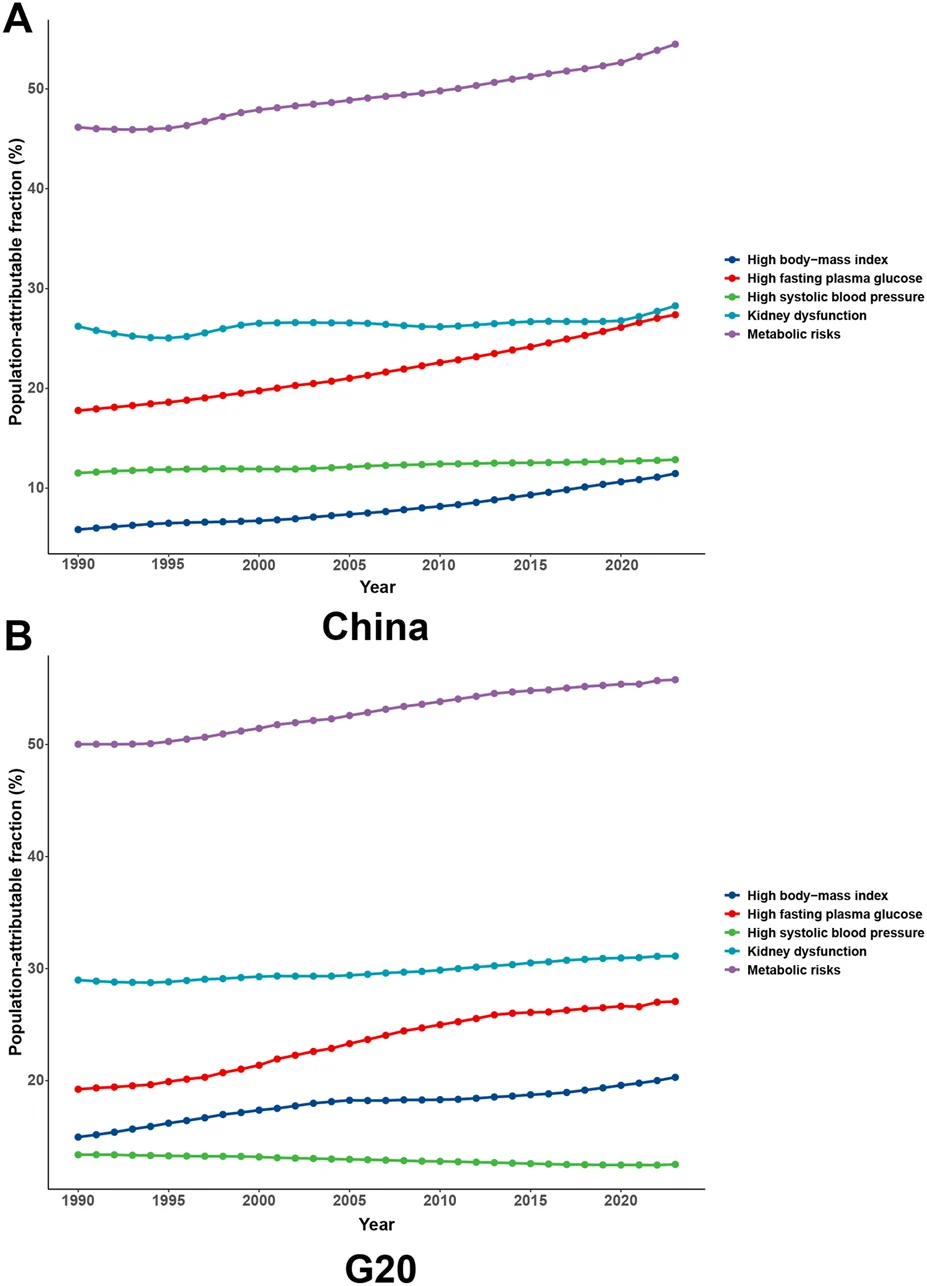
Population-attributable fraction of disability-adjusted life years (DALYs) for peripheral arterial disease attributable to specific metabolic risk factors in China **(A)** and G20 countries **(B)** from 1990 to 2023. (Chongqing, China. 2026).

### Specific metabolic risk factor-attributable PAD burden by age

Across all age groups, a similar pattern for PAFs was observed in China and the G20 countries (Figure 4). The overall PAFs for metabolic risks increased steadily with advancing age in 2023. Among the specific metabolic risk factors, kidney dysfunction and high fasting plasma glucose showed an overall increase in PAFs with advancing age. High systolic blood pressure remained relatively stable across age groups, whereas high body-mass index showed an overall decline with advancing age. Similar trends were observed for both sexes (Supplementary Figures 3, 4).

**FIGURE 4:**
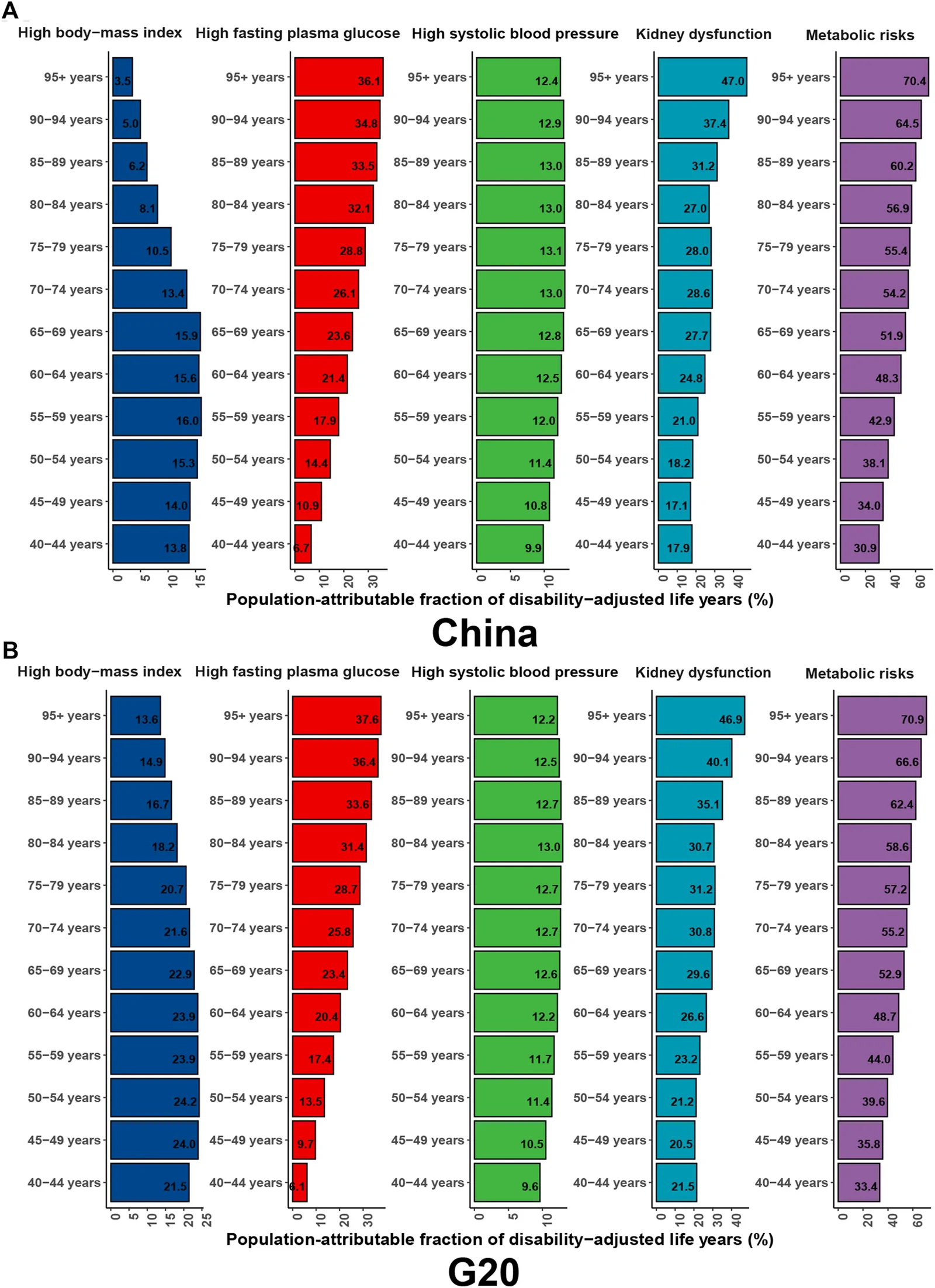
Population-attributable fraction of disability-adjusted life years (DALYs) for peripheral arterial disease attributable to specific metabolic risk factors in China **(A)** and G20 countries **(B)** by age group in 2023. (Chongqing, China. 2026).

### Decomposition analysis

This study quantified the contributions of population growth, aging, and epidemiological changes to the changes in metabolic risk-attributable PAD DALYs from 1990 to 2023 (Supplementary Table 4). Overall, DALYs increased by 68 039 in China and 388 560 in the G20 countries. In China, population growth contributed 65.4%, and aging contributed 46.3%, while epidemiological changes showed a negative contribution (−11.7%). In contrast, for the G20 countries, population growth contributed 104.2%, and aging contributed 34.4%, whereas epidemiological changes had a greater negative contribution (−38.6%).

### ARIMA projections of the PAD burden

Based on the ARIMA model, the DALYs of PAD attributable to metabolic risks in China are projected to continue increasing, reaching 153 890 (95% CI: 132 271 to 179 042) in China by 2035, representing a growth of 53.4% compared with that in 2023 (Supplementary Table 5; Figure 5). Meanwhile, the ASDR initially increases but then decreases, with an overall increase of 1.6% from 2023 to 2035(Supplementary Table 5; Supplementary Figure 5). In contrast, the DALYs in G20 countries are projected to increase by 32% during the same period, whereas the ASDR is projected to decline by 8.8% (Supplementary Table 5; Supplementary Figure 5). Similar trends are observed for both sexes in China and the G20 countries. Although some subgroups still exhibit residual non-stationarity, the ARIMA model generally fits well (Supplementary Tables 6, 7).

**FIGURE 5:**
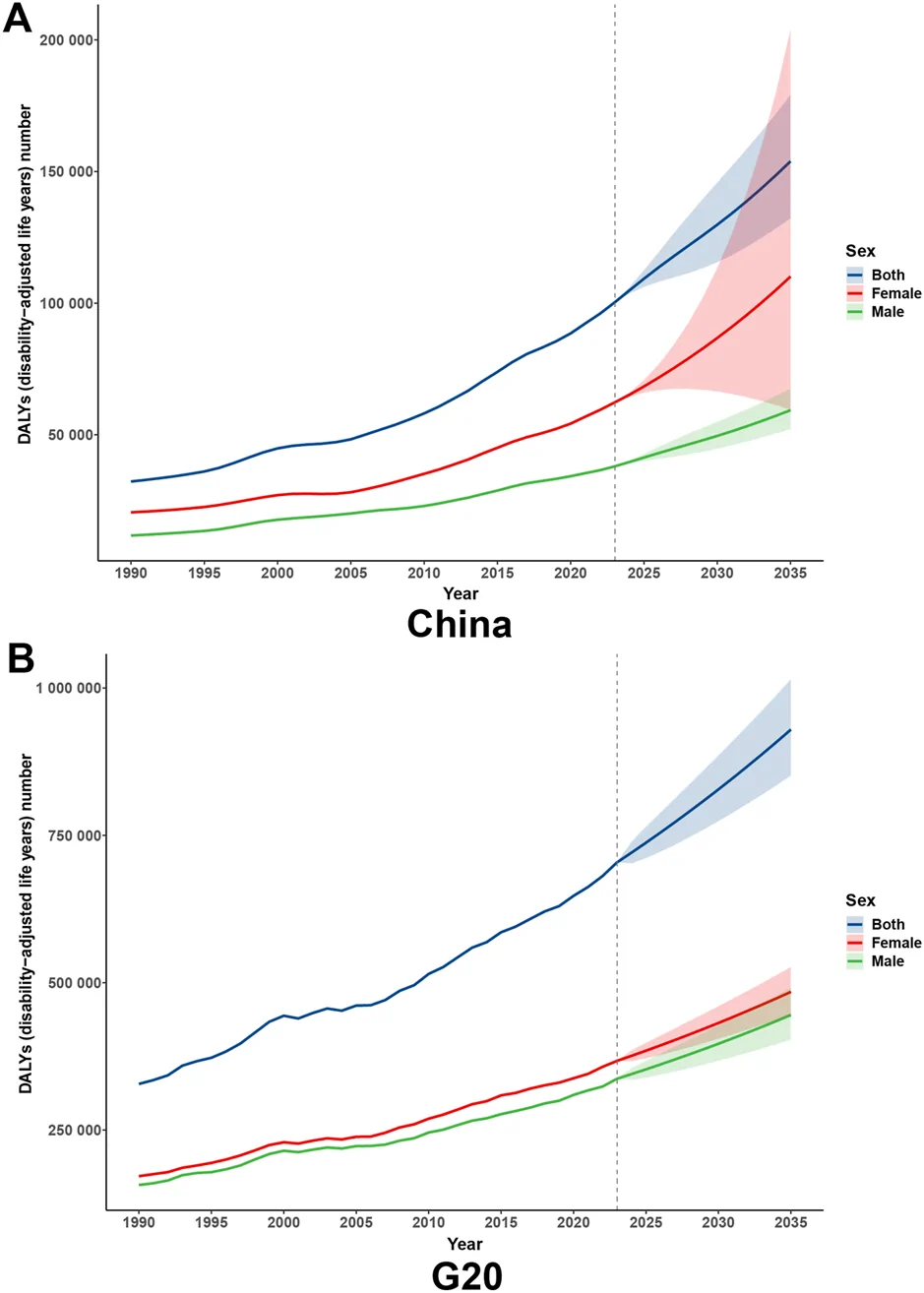
The predicted value of disability-adjusted life years (DALYs) for peripheral arterial disease attributable to metabolic risks in China **(A)** and G20 countries **(B)** from 2024 to 2035. (Chongqing, China. 2026).

## Discussion

This study presents a comprehensive analysis and comparison of the metabolic risk-attributable PAD burden in China and G20 countries from 1990 to 2023, with projections to 2035. Our findings indicated that 54.5% and 55.8% of DALYs of PAD in China and G20 countries, respectively, were attributable to metabolic risks in 2023. These results underscore the critical impact of metabolic risks on PAD, which is consistent with global trends [12]. From 1990 to 2023, the ASDR of PAD attributable to metabolic risks in China and most G20 member countries slightly declined, potentially due to effective prevention and management strategies and advances in medical technology. Notably, the relatively small decline in China may be linked to rapid urbanization, lifestyle changes, and the high prevalence of metabolic diseases in the last decade. Nevertheless, the absolute metabolic risk-attributable DALYs consistently increased in China and all G20 member countries from 1990 to 2023. Decomposition analysis revealed that population growth and aging were the primary drivers. Despite epidemiological changes (potentially due to advances in risk factor management, preventive measures, medical technology, and healthcare accessibility) partially offsetting this effect, though not enough to reverse the overall increasing trend. During the same period, China’s DALYs increased by a remarkable 210.9%, nearly doubling the G20 average. Population aging has contributed more significantly in China, aligning with the country’s more severe aging situation [9].

Additionally, the metabolic risk-attributable PAD burden in China exhibited significant sex and age disparities, differing from the distribution pattern observed in G20 countries. In China, females bore a significantly greater PAD burden than males. This greater burden may be related to biological differences, greater comorbidity loads, healthcare accessibility, and social behavioral factors, warranting further investigation [2, 28]. Overall, the metabolic risk-attributable burden increased with advancing age, likely due to the long-term accumulation of metabolic risks. Moreover, vascular aging and cellular senescence are key drivers of atherosclerosis, which is a complex and irreversible process that accumulates with advancing age [29–31]. The unique physiological changes and multimorbidity characteristics among older adults complicate the management of PAD. Among older females in particular, diminished estrogen protection, high metabolic syndrome prevalence, and delayed healthcare-seeking ultimately result in a greater disease burden [32, 33]. These findings emphasize the importance of age- and sex-tailored screening protocols, particularly for high-risk populations with multiple metabolic comorbidities.

Regarding the composition of metabolic risk factors, kidney dysfunction, high fasting plasma glucose, high body-mass index, and high systolic blood pressure are the specific metabolic risk factors for PAD, which is consistent with the epidemiological characteristics of PAD [2, 34]. Chronic kidney disease (CKD) shares common pathophysiological features with PAD, including inflammation and oxidative stress, significantly accelerating vascular aging [35, 36]. The incidence of PAD in patients with end-stage kidney disease is approximately 4–6 times higher than that in the general population, with the mortality rate doubling [37]. The absolute number of CKD cases has continued to rise from 1990 to 2021, reaching 674 million in 2021 [38]. Diabetes is a recognized risk factor for PAD, and is associated with major adverse limb events and major adverse cardiovascular events [1, 2, 39]. Its age-standardized prevalence rate has increased by 71.5% in China and 90.4% globally over the past three decades [40, 41]. Overweight and obesity are causing a global epidemic trend, with 402 million adults in China being affected by overweight and obesity, ranking first worldwide (2.11 billion adults) [42]. Hypertension accelerates the progression of atherosclerosis by affecting the morphology and function of endothelial cells, smooth muscle cells, and even perivascular adipose tissue, with its burden being particularly pronounced in Asia [43, 44].

Notably, the attributable burdens of high fasting plasma glucose and high body-mass index in China have increased more markedly over the past three decades than those in the G20 countries, becoming the main drivers of the increasing burden of PAD. When stratified by age, the contribution patterns varied across specific metabolic risk factors, highlighting the importance of developing age-differentiated prevention and intervention strategies. Middle-aged individuals may benefit from weight management, whereas older adults require integrated monitoring of glucose and kidney function.

Projections based on the ARIMA model indicate a continuing increase in the absolute PAD burden attributable to metabolic risks by 2035, particularly in China, emphasizing the urgent need for strategic healthcare planning and resource allocation. Based on the above findings and clinical guidelines, we suggest developing targeted strategies at three levels. At the clinical level, early screening and comprehensive management should be strengthened for older adults, particularly those with multiple metabolic diseases. At the public health level, promoting healthy lifestyle interventions and controlling metabolic risk factors should be prioritized. At the policy level, a chronic disease prevention and control system adapted to the characteristics of an aging population should be established, and the allocation of medical resources should be optimized.

Nevertheless, it is important to acknowledge the potential limitations of this study. First, GBD data, derived from national reports and published literature, may raise concerns regarding accuracy and completeness. Second, PAD was defined as an ABI ≤0.90 in GBD 2023. This definition excluded PAD cases that use different ABI cutoffs or lacked available ABI values, which may have resulted in an underestimation of the actual burden. Third, the GBD 2023 lacks PAD data for individuals under 40 years of age. Given the trend toward younger onset of cardiovascular diseases [45, 46], it is reasonable to infer that PAD may be present in younger populations. Fourth, as China is one of the G20 members and retained in the pooled G20 estimates, China’s large population size may exert substantial weight-driven influence on pooled G20-level indicators when comparing China with overall G20 estimates. Fifth, sex-specific and both-sex ARIMA models were fitted independently, which may lead to numerical inconsistency between summed sex-specific forecasts and the aggregated both-sex projection. In addition, the residual non-stationarity present in certain subgroups necessitates cautious interpretation of long-term forecasting results. Finally, while GBD 2023 provides data on the PAD burden attributable to metabolic risks, it does not clarify the interactions among various risk factors. This may result in the overestimation or misinterpretation of individual population-attributable fractions. Future research should focus on establishing a more comprehensive PAD registry system, exploring interactions among various metabolic risk factors, and evaluating the impact of specific interventions (e.g., lifestyle programs or pharmacological treatments).

In summary, this study reveals a substantial and growing absolute PAD burden attributable to metabolic risks in China and G20 countries, with China exhibiting a distinct epidemiological pattern. Projections by 2035 indicate continued escalation of this burden. These findings may provide critical evidence for clinicians and policymakers to prioritize targeted prevention and intervention strategies that address the dual challenges of population aging and metabolic risks.
