# Supplementary material for: Burden trends and disparities of metabolic risk-attributable peripheral arterial disease in China (1990–2035): a comparative analysis with G20 countries
Source: Int J Public Health. 2026 Sep 15;71:1610164. doi: 10.3389/ijph.2026.1610164 (PMC13619535; doi:10.3389/ijph.2026.1610164)
Supplement: Supplementary file 1 [file DataSheet1.docx]

Supplementary File

# 1 Supplementary Data

## 1.1 Age-standardized rate (ASR)

ASR represents the weighted average of age-specific rate and eliminates the confounding effect of differences in age distribution between populations. It was based on the GBD standard population structure, and the standard population age weights used in this study were downloaded directly from the Institute for Health Metrics and Evaluation (IHME) resources. It was expressed per 100 000 population and calculated by the following formula:

$$\text{ASR}=\frac{\sum_{i=1}^{N} a_{i}w_{i}}{\sum_{i=1}^{N} w_{i}}$$

($a_{i}$ represents the age-specific rate in the i^th^ age group, $w_{i}$ represents the number of individuals in the corresponding age group of the GBD standard population, and N represents its total number of individuals).

## 1.2 Average annual percentage change (AAPC)

AAPC is computed as a weighted average of the annual percentage changes from the joinpoint model, with the weights equal to the length of the annual percentage change interval. It is a summary measure of the trend within a prespecified fixed interval, which is the weighted average of the APC within the interval. It uses a single number to describe the average annual percentage change across multiple years and remains valid even if the trend changes during this period. It is calculated by the following formula:

$$\text{AAPC}=\left\{ \exp\left( \frac{\sum w_{i}b_{i}}{\sum w_{i}} \right)-1 \right\}\times100$$

($b_{i}$ represents the slope coefficient for the i^th^ segment with i indexing the segments in the desired range of years, and $w_{i}$ represents the length of each segment in the range of years).

## 1.3 Risk factor definitions

High fasting plasma glucose: Serum fasting plasma glucose >4.9-5.3 mmol/L in adults >25 years of age.

High systolic blood pressure: Brachial systolic blood pressure >105-115 mmHg in adults >25 years of age.

High body mass index: body mass index >20-22.5 kg/m2 in adults >20 years of age.

Kidney dysfunction: Estimated glomerular filtration rate ≤60 mL/min/1.73 m^2^.

## 1.4 Decomposition Analysis

Decomposition analyses can provide quantitative insights into the specific causes that influence the changes of metric numbers over the defined time periods at the global, regional and national levels. Changes in metabolic risk-attributable DALYs of lower extremity peripheral arterial disease were decomposed into population growth, aging, and epidemiological changes. The decomposition results include both the absolute and relative contributions of each factor. Absolute contribution represents the number of deaths or DALYs attributable to the disease, while relative contribution (i.e., the “attribution ratio”) is calculated by dividing the number of attributable deaths or DALYs by the total number of deaths or DALYs in 1990 and multiplying by 100%. This ratio may exceed 100% if the number of attributable deaths surpasses the total number of deaths in 1990. It is calculated by the following formula:

$$A_{a,g,e,t}=\sum_{k=1}^{7} a_{k,t}\times p_{t}\times e_{k,t}$$

($A_{a,g,e,t}$ represents DALYs number accumulated by population growth, aging, and epidemiological changes in year t, $a_{k,t}$ is the proportion of population for the age group k at year t, $p_{t}$ is the population size at year t, and $e_{k,t}$ is represented by DALYs rate for a specific age group k at year t).

## 1.5 Autoregressive integrated moving average (ARIMA) model

### 1.5.1 Model establishment

The autoregressive integrated moving average (ARIMA) model is a widely used time series analysis method that combines autoregression (AR), integration (I), and moving average (MA) components. In the ARIMA (p, d, q) model, “p” represents the number of autoregressive terms, “d” represents the number of differences, and “q” represents the number of moving average terms. We first applied the differencing method to stabilize the time series data and used the Kwiatkowski-Phillips-Schmidt-Shin (KPSS) test to confirm stationarity. Then, a Q-Q plot was utilized to assess whether the residuals followed a normal distribution. Next, we compared the goodness-of-fit of different models using the Akaike information criterion (AIC) and Bayesian information criterion (BIC), selecting the model with the smallest criterion value. Finally, the Ljung-Box test was used to verify the robustness of the residuals. When the residuals of the ARIMA model exhibit randomness (white noise), the model is considered the best linear predictor for short-term time series forecasting.

### 1.5.2 Model assessment

Disability-adjusted life years (Supplementary table 1)

China: Males and Both use ARIMA (2,1,0), while females use ARIMA (0,2,2). The differencing terms (d=1 or 2) address non-stationarity, and AR/MA components capture autocorrelation and moving average patterns. Low AIC and BIC values (e.g., males: -234.9) support model parsimony and goodness-of-fit. All groups have KPSS statistics near 1 (males: 0.946, females: 0.936, both: 0.944) with p-values of 0.01, below common thresholds (e.g., 0.05). This suggests potential non-stationarity in the original series. Although differencing mitigates this, the results caution against overreliance on long-term forecasts. Ljung-Box test p-values exceed 0.05 for all groups (males: 0.824, females: 0.477, both: 0.582), indicating no significant residual autocorrelation and adequate model specification. In conclusion, the ARIMA model performs reliably for short-term predictions, but KPSS-based non-stationarity concerns necessitate external validation. Sensitivity analyses are recommended to account for potential structural shifts.

G20 countries: All sex groups are fitted with an ARIMA (0,1,0) model, indicating a random walk process with unit root and non-stationarity. Low AIC and BIC values (e.g., male AIC=-183.59, BIC=-180.60) suggest a parsimonious and well-fitted model. KPSS statistics are approximately 0.95 with p-values=0.01 for all groups, rejecting the null hypothesis of stationarity. This aligns with d=1 (first differencing), confirming the model’s ability to handle trends effectively. For males, Ljung-Box test statistic=9.50, p-value=0.39; for the total population, Ljung-Box test statistic=13.98, p-value=0.12—both above 0.05, indicating no residual autocorrelation and adequate model capture of data patterns. However, for females, Ljung-Box test statistic=18.07, p-value=0.03, slightly below 0.05, suggesting potential mild residual autocorrelation, though the model remains generally acceptable. In summary, the ARIMA (0,1,0) model provides a good fit for males and the total population, with minor limitations for females. It is suitable for forecasting, but future studies could enhance accuracy by incorporating seasonal adjustments or more complex models.

Age-standardized rate (Supplementary table 2)

China: The ARIMA parameters indicate ARIMA (0,1,3) for males and ARIMA (2,1,0) for females and the both groups. Low AIC and BIC values (males: -238.34/-232.36; females: -256.45/-251.96; both: -256.70/-252.21) suggest model parsimony and high goodness-of-fit. In KPSS tests, p-values for males, females, and both are 0.033, 0.018, and 0.01, respectively, all below the 0.05 significance level, indicating mild non-stationarity, though the differencing order (d=1) partially addresses this. Ljung-Box test p-values are well above 0.05 (males: 0.798; females: 0.463; both: 0.809), confirming no residual autocorrelation and adequate capture of series dynamics. In conclusion, the model fits well, but long-term forecasts should be interpreted cautiously due to slight non-stationarity.

G20 countries: The ARIMA model parameters are (1,1,0) for all sex groups, indicating first-order differencing to remove trends and reliance on a first-order autoregressive term. Diagnostic metrics show low AIC and BIC values (Male: -185.75/-181.26; Female: -203.66/-199.17; Both: -195.46/-190.97), supporting model parsimony and goodness-of-fit. However, KPSS test statistics exceed critical values (approximately 0.91, p=0.01), rejecting the null hypothesis of stationarity and suggesting potential unresolved long-term trends in residuals, though first-order differencing partially addresses this. Ljung-Box test p-values are all above 0.05 (Male: 0.28; Female: 0.11; Both: 0.14), indicating no significant autocorrelation in residuals and adequate model specification. In summary, while the model is not perfectly stationary, it offers acceptable predictive performance, particularly for short-term public health planning.

# 2 Supplementary Figures and Tables

## 2.1 Supplementary Figures

Supplementary Figure 1. Population-attributable fraction of disability-adjusted life years (DALYs) for peripheral arterial disease attributable to specific metabolic risk factors among males in China (A) and G20 countries (B), from 1990 to 2023.

Supplementary Figure 2. Population-attributable fraction of disability-adjusted life years (DALYs) for peripheral arterial disease attributable to specific metabolic risk factors among females in China (A) and G20 countries (B), from 1990 to 2023.

Supplementary Figure 3. Population-attributable fraction of disability-adjusted life years (DALYs) for peripheral arterial disease attributable to specific metabolic risk factors among males in China (A) and G20 countries (B) by age group, in 2023.

Supplementary Figure 4. Population-attributable fraction of disability-adjusted life years (DALYs) for peripheral arterial disease attributable to specific metabolic risk factors among females in China (A) and G20 countries (B) by age group, in 2023.

Supplementary Figure 5. The predicted value of age-standardized rate for peripheral arterial disease attributable to metabolic risks among older adults in China (A) and G20 countries (B) from 2024 to 2035.

## 2.2 Supplementary Tables

Supplementary Table 1: Number and age-standardized rate (ASR) of Disability-adjusted life years (DALYs) for lower extremity peripheral arterial disease attributable to metabolic risks in 1990/2023, and corresponding average annual percentage change (AAPC) from 1990 to 2023.

| **Location** | **Number** | | | **ASR per 100000** | | |
| --- | --- | --- | --- | --- | --- | --- |
|  | **1990 (95% UI)** | **2023 (95% UI)** | **1990-2023 AAPC (95%CI)** | **1990 (95% UI)** | **2023 (95% UI)** | **1990-2023 AAPC (95%CI)** |
| China | 32259 (16934, 57802) | 100298 (51116, 184384) | 3.55 (3.46, 3.65) | 4.98 (2.56, 8.91) | 4.41 (2.26, 8.09) | -0.35 (-0.46, -0.25) |
| Canada | 6187 (4382, 7795) | 12628(9330,16870) | 1.98 (1.81, 2.15) | 19.20 (13.65, 24.23) | 14.56 (10.77, 19.39) | -0.74 (-0.92, -0.55) |
| Argentina | 1864 (1165, 2752) | 4779 (3321, 6702) | 3.07 (2.70, 3.43) | 6.34 (3.98, 9.27) | 7.20 (5.00, 10.15) | 0.61 (0.28, 0.93) |
| Türkiye | 2839 (1666, 4452) | 11479 (7966, 15852) | 4.35 (4.03, 4.66) | 9.46 (5.67, 14.20) | 12.25 (8.58, 16.92) | 0.75 (0.45, 1.04) |
| Australia | 4658 (3347, 5803) | 7585 (5550, 9752) | 1.62 (1.14, 2.10) | 25.24 (18.21, 31.62) | 12.84 (9.36, 16.67) | -2.05 (-2.36, -1.74) |
| Germany | 27785 (19682, 38131) | 49878 (36841, 66355) | 2.26 (1.84, 2.69) | 20.64 (14.72, 28.11) | 21.65 (16.09, 28.59) | 0.51 (0.11, 0.90) |
| Brazil | 12869 (9253, 17492) | 44473 (33242, 61234) | 3.98 (3.68, 4.29) | 17.00 (12.21, 23.27) | 16.99 (12.67, 23.41) | 0.10 (-0.21, 0.42) |
| France | 8683 (5936, 12215) | 12251 (8345, 17935) | 0.90 (0.43, 1.36) | 9.07 (6.12, 12.76) | 6.87 (4.65, 10.30) | -1.02 (-1.33, -0.71) |
| India | 26899 (14364, 44987) | 109193 (64899, 180287) | 4.34 (4.24, 4.45) | 8.35 (4.36, 14.24) | 10.37 (6.18, 16.82) | 0.70 (0.62, 0.79) |
| Indonesia | 3326 (1650, 5975) | 12389 (6369, 21210) | 4.08 (4.00, 4.16) | 4.48 (2.15, 8.23) | 5.75 (2.93, 10.08) | 0.77 (0.73, 0.80) |
| Mexico | 5985 (4257, 8687) | 14673 (9293, 23390) | 2.86 (2.65, 3.06) | 15.79 (11.18, 23.00) | 11.29 (7.17, 17.98) | -0.99 (-1.20, -0.78) |
| Italy | 16418 (11421, 21880) | 20449 (14577 ,29308) | 0.88 (0.50, 1.27) | 18.45 (12.91, 24.59) | 11.01 (7.76, 15.54) | -1.53 (-1.72, -1.34) |
| Japan | 8656 (5335, 13012) | 27747 (17672, 41346) | 3.58 (3.28, 3.89) | 5.51 (3.42, 8.24) | 5.17 (3.38, 7.76) | -0.11 (-0.37, 0.16) |
| Republic of Korea | 1161 (703, 1693) | 4824 (3271, 7328) | 4.38 (4.14, 4.62) | 5.31 (3.20, 7.99) | 4.73 (3.22, 7.18) | -0.28 (-0.47, -0.09) |
| Russian Federation | 71687 (48792, 95005) | 82333 (55440, 109208) | 0.25 (0.01, 0.49) | 43.23 (29.39, 57.07) | 32.06 (21.65, 42.43) | -1.01 (-1.24, -0.78) |
| Saudi Arabia | 187 (100, 322) | 445 (247, 745) | 2.67 (2.58, 2.77) | 4.85 (2.49, 8.56) | 4.64 (2.48, 8.06) | -0.10 (-0.19, -0.00) |
| South Africa | 4035 (2696, 5785) | 13909 (10021, 18528) | 3.64 (3.35, 3.93) | 21.09 (14.06, 29.99) | 27.77 (19.71, 37.39) | 0.87 (0.52, 1.22) |
| United Kingdom | 28587 (21097, 37407) | 35704 (25462, 46666) | 0.80 (0.41, 1.19) | 30.26 (22.55, 39.29) | 22.20 (15.76, 29.37) | -0.82 (-1.10, -0.55) |
| United States of America | 64068 (45791, 83014) | 139178 (106822, 177342) | 2.35 (2.19, 2.50) | 19.27 (13.85, 24.85) | 21.79 (16.73, 27.53) | 0.31 (0.17, 0.45) |
| European Union | 124950 (93453, 159786) | 181761 (137446, 241787) | 1.20 (0.92, 1.48) | 20.37 (15.31, 26.04) | 15.80 (11.90, 20.90) | -0.80 (-1.06, -0.55) |

Supplementary Table 2. Number and age-standardized rate (ASR) of disability-adjusted life years for peripheral arterial disease attributable to metabolic risks in China and G20 countries by year and sex.

| **Year** | **Male** | | **Female** | |
| --- | --- | --- | --- | --- |
|  | **Number (95% UI)** | **ASR per 100000 (95% UI)** | **Number (95% UI)** | **ASR per 100000 (95% UI)** |
| **China** | | | | |
| 1990 | 11727 (6495, 20469) | 3.96 (2.21, 6.97) | 20531 (9947, 37592) | 5.69 (2.75, 10.30) |
| 1991 | 12040 (6724, 20828) | 3.93 (2.20, 6.93) | 20833 (10064, 38039) | 5.60 (2.69, 10.11) |
| 1992 | 12356 (6954, 21372) | 3.90 (2.15, 6.86) | 21184 (10299, 38801) | 5.52 (2.67, 10.01) |
| 1993 | 12693 (7172, 22021) | 3.89 (2.17, 6.86) | 21569 (10447, 39415) | 5.47 (2.63, 9.87) |
| 1994 | 13082 (7220, 22784) | 3.88 (2.10, 6.87) | 22037 (10617, 40070) | 5.44 (2.61, 9.77) |
| 1995 | 13485 (7450, 23680) | 3.87 (2.09, 6.91) | 22577 (10939, 40736) | 5.42 (2.62, 9.67) |
| 1996 | 14103 (7654, 25120) | 3.92 (2.15, 6.99) | 23315 (11352, 42005) | 5.44 (2.64, 9.70) |
| 1997 | 15011 (7961, 26893) | 4.04 (2.20, 7.19) | 24245 (11715, 43818) | 5.51 (2.66, 9.85) |
| 1998 | 16046 (8744, 28833) | 4.19 (2.28, 7.47) | 25253 (12072, 45667) | 5.59 (2.69, 10.00) |
| 1999 | 17036 (9051, 30925) | 4.30 (2.32, 7.72) | 26244 (12488, 47789) | 5.66 (2.71, 10.19) |
| 2000 | 17748 (9202, 31993) | 4.33 (2.33, 7.73) | 27090 (12822, 49249) | 5.68 (2.73, 10.22) |
| 2001 | 18245 (9342, 33317) | 4.29 (2.27, 7.71) | 27550 (13031, 50799) | 5.61 (2.69, 10.24) |
| 2002 | 18669 (9532, 34169) | 4.24 (2.22, 7.68) | 27634 (13098, 51117) | 5.45 (2.62, 10.00) |
| 2003 | 19088 (9626, 35145) | 4.17 (2.17, 7.63) | 27522 (13155, 51097) | 5.26 (2.54, 9.65) |
| 2004 | 19587 (10089, 36047) | 4.12 (2.13, 7.53) | 27627 (13217, 51729) | 5.09 (2.45, 9.38) |
| 2005 | 20108 (10537, 37255) | 4.07 (2.10, 7.44) | 28171 (13485, 52984) | 5.00 (2.40, 9.25) |
| 2006 | 20797 (10919, 39107) | 4.03 (2.11, 7.34) | 29297 (14037, 55105) | 5.00 (2.41, 9.26) |
| 2007 | 21365 (11404, 40026) | 3.97 (2.11, 7.14) | 30565 (14685, 57777) | 5.01 (2.42, 9.35) |
| 2008 | 21753 (11744, 40094) | 3.87 (2.09, 6.89) | 32002 (15577, 60545) | 5.04 (2.48, 9.42) |
| 2009 | 22260 (12188, 40860) | 3.79 (2.08, 6.79) | 33592 (16369, 62830) | 5.07 (2.51, 9.43) |
| 2010 | 22956 (12573, 41756) | 3.75 (2.06, 6.69) | 35177 (17248, 66035) | 5.09 (2.50, 9.51) |
| 2011 | 23898 (13016, 42896) | 3.74 (2.03, 6.69) | 36798 (18110, 68797) | 5.10 (2.51, 9.48) |
| 2012 | 25012 (13434, 44901) | 3.75 (2.01, 6.70) | 38666 (18937, 72652) | 5.13 (2.51, 9.58) |
| 2013 | 26114 (13753, 46873) | 3.75 (1.96, 6.72) | 40606 (19918, 76553) | 5.16 (2.52, 9.64) |
| 2014 | 27499 (14761, 49288) | 3.77 (1.99, 6.76) | 42910 (21177, 81058) | 5.20 (2.56, 9.78) |
| 2015 | 28820 (15649, 50969) | 3.79 (2.03, 6.69) | 45065 (22271, 85727) | 5.23 (2.58, 9.83) |
| 2016 | 30299 (16663, 53207) | 3.82 (2.06, 6.70) | 47262 (23514, 91155) | 5.24 (2.61, 9.99) |
| 2017 | 31597 (17569, 55119) | 3.83 (2.10, 6.63) | 49079 (24590, 94622) | 5.22 (2.61, 10.00) |
| 2018 | 32434 (18093, 56342) | 3.78 (2.08, 6.54) | 50553 (25236, 97665) | 5.16 (2.58, 9.90) |
| 2019 | 33276 (18685, 58765) | 3.70 (2.04, 6.47) | 52237 (26189, 100769) | 5.08 (2.54, 9.73) |
| 2020 | 34279 (19144, 60358) | 3.65 (2.03, 6.42) | 54248 (26980, 104168) | 5.04 (2.51, 9.60) |
| 2021 | 35421 (19842, 61771) | 3.62 (2.03, 6.34) | 56884 (28785, 109272) | 5.04 (2.55, 9.61) |
| 2022 | 36553 (20393, 64158) | 3.60 (2.02, 6.32) | 59459 (30016, 113631) | 5.04 (2.53, 9.57) |
| 2023 | 38007 (20660, 67545) | 3.61 (1.99, 6.41) | 62292 (31083, 117390) | 5.06 (2.55, 9.50) |
| **G20** | | | | |
| 1990 | 156617 (113289, 202994) | 15.97 (11.45, 20.69) | 171538 (116040, 251497) | 12.78 (8.79, 18.66) |
| 1991 | 159634 (116489, 205740) | 15.79 (11.38, 20.52) | 175082 (118598, 256098) | 12.67 (8.70, 18.46) |
| 1992 | 164341 (120369, 210809) | 15.74 (11.50, 20.30) | 178449 (121342, 260808) | 12.56 (8.65, 18.28) |
| 1993 | 173314 (127083, 221367) | 16.05 (11.94, 20.53) | 185847 (127011, 270602) | 12.66 (8.81, 18.36) |
| 1994 | 177084 (128424, 226217) | 15.90 (11.85, 20.34) | 189698 (129706, 275941) | 12.58 (8.75, 18.23) |
| 1995 | 178428 (129721, 228166) | 15.57 (11.61, 20.01) | 194124 (133683, 282406) | 12.49 (8.76, 18.11) |
| 1996 | 183240 (134395, 235146) | 15.53 (11.64, 20.00) | 199799 (138457, 289376) | 12.52 (8.83, 18.08) |
| 1997 | 189831 (139444, 245694) | 15.58 (11.64, 20.05) | 206804 (144558, 299470) | 12.61 (8.93, 18.20) |
| 1998 | 199936 (146914, 259247) | 15.95 (11.85, 20.55) | 215280 (151043, 311379) | 12.78 (9.04, 18.44) |
| 1999 | 209233 (154813, 270808) | 16.19 (12.00, 20.86) | 224421 (157087, 325161) | 12.93 (9.08, 18.65) |
| 2000 | 214774 (158832, 276547) | 16.13 (12.05, 20.98) | 229122 (161381, 331590) | 12.86 (9.11, 18.46) |
| 2001 | 212365 (157598, 277073) | 15.51 (11.72, 20.20) | 226893 (158762, 331452) | 12.39 (8.75, 17.95) |
| 2002 | 216618 (161548, 286337) | 15.34 (11.57, 19.99) | 231901 (162725, 338073) | 12.29 (8.68, 17.80) |
| 2003 | 220314 (164851, 291923) | 15.10 (11.42, 19.74) | 235842 (165586, 344387) | 12.14 (8.57, 17.62) |
| 2004 | 218523 (163242, 289743) | 14.53 (11.00, 19.06) | 233897 (163445, 344432) | 11.77 (8.28, 17.23) |
| 2005 | 222665 (166964, 295401) | 14.36 (10.89, 18.87) | 238461 (166737, 351121) | 11.60 (8.15, 16.99) |
| 2006 | 222870 (168864, 297682) | 13.95 (10.74, 18.39) | 238799 (166126, 353593) | 11.29 (7.90, 16.64) |
| 2007 | 225189 (170248, 301851) | 13.63 (10.50, 18.04) | 245286 (171479, 363807) | 11.23 (7.91, 16.60) |
| 2008 | 231881 (174534, 309610) | 13.56 (10.41, 17.92) | 254298 (179364, 376878) | 11.26 (7.99, 16.64) |
| 2009 | 236108 (177885, 314275) | 13.35 (10.30, 17.66) | 259682 (182814, 387308) | 11.17 (7.90, 16.62) |
| 2010 | 245723 (185604, 327393) | 13.39 (10.34, 17.72) | 269162 (190145, 400429) | 11.17 (7.92, 16.61) |
| 2011 | 250480 (189644, 335580) | 13.16 (10.17, 17.47) | 275975 (194472, 412458) | 11.07 (7.83, 16.57) |
| 2012 | 258350 (197646, 347786) | 13.10 (10.19, 17.49) | 284648 (200445, 427408) | 11.02 (7.79, 16.59) |
| 2013 | 265748 (203858, 358934) | 13.01 (10.10, 17.42) | 293687 (207868, 443084) | 11.01 (7.81, 16.66) |
| 2014 | 269871 (206540, 369220) | 12.74 (9.85, 17.29) | 298873 (212149, 451466) | 10.86 (7.73, 16.50) |
| 2015 | 276819 (211168, 377757) | 12.60 (9.74, 17.14) | 308734 (220877, 466131) | 10.81 (7.74, 16.43) |
| 2016 | 282184 (213458, 383956) | 12.40 (9.53, 16.85) | 312896 (222447, 473653) | 10.65 (7.57, 16.19) |
| 2017 | 287958 (216128, 391314) | 12.22 (9.31, 16.63) | 320112 (226141, 485264) | 10.52 (7.43, 16.02) |
| 2018 | 295060 (219669, 401008) | 12.10 (9.18, 16.45) | 325786 (228277, 493765) | 10.38 (7.27, 15.80) |
| 2019 | 299611 (222910, 410871) | 11.85 (8.87, 16.20) | 330310 (228835, 502120) | 10.19 (7.05, 15.55) |
| 2020 | 309594 (230748, 419681) | 11.83 (8.93, 15.99) | 337805 (234167, 511610) | 10.12 (7.00, 15.37) |
| 2021 | 317212 (235701, 428001) | 11.75 (8.81, 15.85) | 345234 (236726, 524957) | 10.06 (6.89, 15.33) |
| 2022 | 323748 (239206, 435343) | 11.64 (8.67, 15.56) | 357190 (242793, 539406) | 10.05 (6.80, 15.24) |
| 2023 | 336944 (247587, 449284) | 11.73 (8.63, 15.58) | 367269 (248366, 552747) | 10.03 (6.76, 15.15) |

Supplementary Table 3. Number and age-standardized rate (ASR) of disability-adjusted life years for peripheral arterial disease attributable to metabolic risks in China and G20 countries by age group and sex, in 2023.

| **Age group** | **Male** | | **Female** | |
| --- | --- | --- | --- | --- |
|  | **Number (95% UI)** | **ASR per 100000 (95% UI)** | **Number (95% UI)** | **ASR per 100000 (95% UI)** |
| **China** | | | | |
| 40-44 years | 87 (46, 138) | 0.19 (0.10, 0.29) | 50 (25, 85) | 0.11 (0.06, 0.19) |
| 45-49 years | 181 (102, 277) | 0.36 (0.20, 0.55) | 90 (50, 147) | 0.18 (0.10, 0.30) |
| 50-54 years | 536 (323, 846) | 0.89 (0.54, 1.41) | 471 (249, 976) | 0.81 (0.43, 1.67) |
| 55-59 years | 1656 (862, 2998) | 2.81 (1.46, 5.09) | 1939 (933, 4117) | 3.27 (1.57, 6.94) |
| 60-64 years | 3115 (1601, 6073) | 7.69 (3.95, 14.98) | 3870 (1662, 8198) | 9.46 (4.06, 20.04) |
| 65-69 years | 6349 (3034, 12199) | 16.84 (8.05, 32.36) | 8397 (3548, 17357) | 21.75 (9.19, 44.95) |
| 70-74 years | 8095 (3749, 15368) | 27.60 (12.78, 52.40) | 12352 (5214, 25094) | 38.93 (16.43, 79.09) |
| 75-79 years | 6677 (3158, 13415) | 38.75 (18.33, 77.86) | 11523 (4930, 23730) | 58.92 (25.21, 121.34) |
| 80-84 years | 5236 (2470, 9879) | 52.03 (24.55, 98.18) | 10050 (4462, 19960) | 81.03 (35.98, 160.94) |
| 85-89 years | 3675 (1781, 6334) | 71.98 (34.89, 124.05) | 7884 (3694, 14342) | 107.03 (50.14, 194.7) |
| 90-94 years | 1773 (916, 3361) | 104.14 (53.83, 197.41) | 4082 (2049, 7940) | 144.44 (72.50, 280.97) |
| 95+ years | 627 (295, 1243) | 147.97 (69.72, 293.26) | 1584 (733, 3192) | 193.65 (89.67, 390.28) |
| **G20** | | | | |
| 40-44 years | 1795 (1155, 2428) | 1.10 (0.71, 1.49) | 1086 (683, 1493) | 0.69 (0.43, 0.95) |
| 45-49 years | 3255 (2208, 4276) | 2.12 (1.44, 2.78) | 1806 (1187, 2482) | 1.20 (0.79, 1.64) |
| 50-54 years | 7989 (5685, 10166) | 5.18 (3.69, 6.59) | 4204 (2903, 5827) | 2.75 (1.90, 3.81) |
| 55-59 years | 17203 (12512, 22531) | 12.10 (8.80, 15.85) | 10380 (6910, 15610) | 7.11 (4.73, 10.69) |
| 60-64 years | 35580 (25783, 46572) | 31.04 (22.50, 40.64) | 22529 (14305, 35695) | 18.72 (11.89, 29.66) |
| 65-69 years | 54143 (39286, 74508) | 55.24 (40.08, 76.02) | 40700 (23738, 68600) | 38.53 (22.47, 64.94) |
| 70-74 years | 61234 (43186, 84968) | 80.91 (57.06, 112.27) | 55897 (32571, 91637) | 65.24 (38.02, 106.95) |
| 75-79 years | 53922 (37921, 78082) | 111.87 (78.67, 161.99) | 58710 (37297, 97607) | 102.08 (64.85, 169.71) |
| 80-84 years | 44184 (31790, 62365) | 152.94 (110.04, 215.87) | 57634 (38074, 90210) | 148.98 (98.42, 233.18) |
| 85-89 years | 32617 (23951, 43845) | 224.69 (164.99, 302.03) | 52463 (35194, 74685) | 227.71 (152.76, 324.17) |
| 90-94 years | 17998 (13418, 24752) | 358.13 (267.01, 492.53) | 38362 (27101, 54082) | 389.46 (275.14, 549.06) |
| 95+ years | 7025 (5289, 9668) | 546.49 (411.46, 752.08) | 23498 (16963, 32115) | 706.04 (509.70, 964.96) |

Supplementary Table 4. Changes in disability-adjusted life years of peripheral arterial disease attributable to metabolic risks according to population growth, aging, and epidemiological changes from 1990 to 2023 in China and G20 countries.

| **Location** | **Overall difference** | **Population growth** | **Aging** | **Epidemiological changes** | **Population growth Percentage (%)** | **Aging Percentage (%)** | **Epidemiological changes Percentage (%)** |
| --- | --- | --- | --- | --- | --- | --- | --- |
| China | 68039 | 44509 | 31478 | -7948 | 65.4 | 46.3 | -11.7 |
| G20 | 388560 | 405014 | 133645 | -150099 | 104.2 | 34.4 | -38.6 |

Supplementary Table 5. Number and age-standardized rate (ASR) of disability-adjusted life years for peripheral arterial disease attributable to metabolic risks in China and G20 countries based on the autoregressive integrated moving average (ARIMA) model, from 2024 to 2035.

| **Year** | **Both** | | **Male** | | **Female** | |
| --- | --- | --- | --- | --- | --- | --- |
|  | **Number (95% CI)** | **ASR per 100000 (95% CI)** | **Number (95% CI)** | **ASR per 100000 (95% CI)** | **Number (95% CI)** | **ASR per 100000 (95% CI)** |
| **China** | | | | | | |
| 2024 | 104813 (103658, 105981) | 4.44 (4.40, 4.48) | 39654 (39156, 40159) | 3.65 (3.61, 3.70) | 65180 (64381, 65989) | 5.10 (5.06, 5.15) |
| 2025 | 109291 (106217, 112453) | 4.48 (4.38, 4.59) | 41367 (40098, 42676) | 3.71 (3.59, 3.83) | 68363 (66063, 70742) | 5.14 (5.02, 5.27) |
| 2026 | 113592 (108128, 119333) | 4.52 (4.33, 4.71) | 43057 (40904, 45323) | 3.73 (3.53, 3.94) | 71701 (66997, 76734) | 5.17 (4.96, 5.39) |
| 2027 | 117702 (109737, 126245) | 4.53 (4.27, 4.81) | 44694 (41699, 47905) | 3.73 (3.46, 4.01) | 75202 (67438, 83859) | 5.18 (4.88, 5.51) |
| 2028 | 121695 (111376, 132970) | 4.53 (4.20, 4.89) | 46298 (42580, 50342) | 3.73 (3.41, 4.08) | 78873 (67467, 92208) | 5.18 (4.80, 5.59) |
| 2029 | 125689 (113281, 139455) | 4.52 (4.14, 4.93) | 47914 (43598, 52656) | 3.73 (3.36, 4.13) | 82724 (67133, 101936) | 5.17 (4.72, 5.65) |
| 2030 | 129799 (115575, 145775) | 4.50 (4.09, 4.96) | 49586 (44767, 54923) | 3.73 (3.33, 4.18) | 86763 (66477, 113240) | 5.15 (4.67, 5.67) |
| 2031 | 134115 (118285, 152063) | 4.49 (4.05, 4.97) | 51347 (46074, 57223) | 3.73 (3.29, 4.22) | 91000 (65534, 126361) | 5.13 (4.62, 5.69) |
| 2032 | 138682 (121378, 158453) | 4.47 (4.02, 4.98) | 53209 (47493, 59613) | 3.73 (3.26, 4.26) | 95443 (64335, 141592) | 5.12 (4.60, 5.70) |
| 2033 | 143512 (124789, 165045) | 4.47 (4.01, 4.99) | 55171 (48997, 62122) | 3.73 (3.23, 4.30) | 100103 (62911, 159282) | 5.11 (4.58, 5.71) |
| 2034 | 148590 (128442, 171899) | 4.47 (3.99, 5.01) | 57220 (50560, 64756) | 3.73 (3.20, 4.34) | 104990 (61290, 179848) | 5.12 (4.57, 5.73) |
| 2035 | 153890 (132271, 179042) | 4.48 (3.99, 5.03) | 59345 (52167, 67510) | 3.73 (3.18, 4.37) | 110116 (59501, 203788) | 5.13 (4.56, 5.76) |
| **G20** | | | | | | |
| 2024 | 720699 (702691, 739168) | 10.83 (10.58, 11.08) | 344858 (335210, 354784) | 11.67 (11.36, 11.99) | 375840 (366899, 385000) | 9.97 (9.77, 10.18) |
| 2025 | 737570 (711642, 764442) | 10.75 (10.35, 11.17) | 352958 (339074, 367411) | 11.58 (11.09, 12.09) | 384612 (371735, 397934) | 9.90 (9.57, 10.25) |
| 2026 | 754835 (722466, 788655) | 10.67 (10.15, 11.22) | 361248 (343922, 379447) | 11.48 (10.85, 12.14) | 393588 (377511, 410349) | 9.83 (9.40, 10.29) |
| 2027 | 772505 (734382, 812607) | 10.59 (9.96, 11.25) | 369733 (349333, 391323) | 11.37 (10.63, 12.16) | 402773 (383836, 422644) | 9.76 (9.25, 10.31) |
| 2028 | 790589 (747097, 836612) | 10.50 (9.80, 11.26) | 378417 (355151, 403206) | 11.27 (10.44, 12.17) | 412173 (390568, 434973) | 9.69 (9.10, 10.32) |
| 2029 | 809096 (760468, 860833) | 10.42 (9.64, 11.26) | 387305 (361298, 415183) | 11.17 (10.26, 12.16) | 421792 (397634, 447417) | 9.62 (8.97, 10.32) |
| 2030 | 828036 (774415, 885370) | 10.33 (9.50, 11.25) | 396401 (367730, 427308) | 11.06 (10.08, 12.14) | 431635 (404995, 460028) | 9.55 (8.85, 10.31) |
| 2031 | 847420 (788888, 910294) | 10.25 (9.36, 11.23) | 405712 (374421, 439617) | 10.96 (9.92, 12.11) | 441709 (412628, 472839) | 9.48 (8.73, 10.30) |
| 2032 | 867257 (803857, 935657) | 10.17 (9.22, 11.21) | 415241 (381354, 452138) | 10.86 (9.77, 12.08) | 452017 (420517, 485877) | 9.41 (8.62, 10.28) |
| 2033 | 887558 (819304, 961500) | 10.09 (9.10, 11.18) | 424993 (388518, 464893) | 10.76 (9.62, 12.04) | 462566 (428653, 499163) | 9.35 (8.51, 10.26) |
| 2034 | 908335 (835214, 987858) | 10.00 (8.97, 11.15) | 434975 (395905, 477901) | 10.66 (9.47, 12.00) | 473361 (437029, 512714) | 9.28 (8.41, 10.24) |
| 2035 | 929599 (851582, 1014763) | 9.92 (8.86, 11.12) | 445192 (403512, 491177) | 10.57 (9.33, 11.96) | 484408 (445643, 526546) | 9.21 (8.31, 10.21) |

Supplementary Table 6. Diagnostic metrics of the autoregressive integrated moving average (ARIMA) model for the disability-adjusted life years (DALYs) of peripheral arterial disease attributable to metabolic risks

| **Sex** | **p** | **d** | **q** | **AIC** | **BIC** | **KPSS_stat** | **KPSS_pval** | **LB_stat** | **LB_pval** |
| --- | --- | --- | --- | --- | --- | --- | --- | --- | --- |
| **China** | | | | | | | | | |
| Both | 2 | 1 | 0 | -244.73 | -238.75 | 0.94 | 0.01 | 5.64 | 0.58 |
| Male | 2 | 1 | 0 | -234.92 | -228.94 | 0.95 | 0.01 | 3.60 | 0.82 |
| Female | 0 | 2 | 2 | -234.44 | -230.04 | 0.94 | 0.01 | 7.56 | 0.48 |
| **G20** | | | | | | | | | |
| Both | 0 | 1 | 0 | -191.46 | -188.46 | 0.95 | 0.01 | 13.98 | 0.12 |
| Male | 0 | 1 | 0 | -183.59 | -180.60 | 0.95 | 0.01 | 9.50 | 0.39 |
| Female | 0 | 1 | 0 | -194.73 | -191.73 | 0.95 | 0.01 | 18.07 | 0.03 |

Supplementary Table 7. Diagnostic metrics of the autoregressive integrated moving average (ARIMA) model for the age-standardized rate of peripheral arterial disease attributable to metabolic risks.

| **Sex** | **p** | **d** | **q** | **AIC** | **BIC** | **KPSS_stat** | **KPSS_pval** | **LB_stat** | **LB_pval** |
| --- | --- | --- | --- | --- | --- | --- | --- | --- | --- |
| **China** | | | | | | | | | |
| Both | 2 | 1 | 0 | -256.7 | -252.21 | 0.74 | 0.01 | 4.50 | 0.81 |
| Male | 0 | 1 | 3 | -238.34 | -232.36 | 0.54 | 0.03 | 3.84 | 0.80 |
| Female | 2 | 1 | 0 | -256.45 | -251.96 | 0.65 | 0.02 | 7.70 | 0.46 |
| **G20** | | | | | | | | | |
| Both | 1 | 1 | 0 | -195.46 | -190.97 | 0.91 | 0.01 | 12.23 | 0.14 |
| Male | 1 | 1 | 0 | -185.75 | -181.26 | 0.91 | 0.01 | 9.83 | 0.28 |
| Female | 1 | 1 | 0 | -203.66 | -199.17 | 0.91 | 0.01 | 13.15 | 0.11 |
